# Risk assessment of cytologically indeterminate thyroid nodules with integrated molecular testing and repeat biopsy: a surgical decision-oriented tool

**DOI:** 10.1186/s12957-023-02917-x

**Published:** 2023-02-03

**Authors:** Xuhuizi Guan, Tian Yu, Zheng Zhang, Lan Chen, An Yan, Yao Li, Jiankun Li, Dongdong Wang, Jie Sun, Feiliang Wang, Gang Miao

**Affiliations:** 1 The Key Laboratory of Geriatrics, Beijing Institute of Geriatrics, Institute of Geriatric Medicine, Chinese Academy of Medical Sciences, Beijing Hospital/National Center of Gerontology of National Health Commission, Beijing, People’s Republic of China; 2 Department of General Surgery, Beijing Hospital, National Center of Gerontology; Institute of Geriatric Medicine, Chinese Academy of Medical Sciences, NO.1 Da Hua Road, Dong Dan, Beijing, 100730 People’s Republic of China; 3 Department of General Surgery, Peking Union Medical College Hospital, Chinese Academy of Medical Sciences, Peking Union Medical College, Beijing, People’s Republic of China; 4 Department of Ultrasonography, Beijing Hospital, National Center of Gerontology; Institute of Geriatric Medicine, Chinese Academy of Medical Sciences, Beijing, People’s Republic of China; 5 Department of Pathology, Beijing Hospital, National Center of Gerontology; Institute of Geriatric Medicine, Chinese Academy of Medical Sciences, Beijing, People’s Republic of China; 6 Department of Hematology, Peking University People’s Hospital, Peking University Institute of Hematology, National Clinical Research Center for Hematologic Disease, Beijing, People’s Republic of China

**Keywords:** Indeterminate thyroid nodules, Serum testing, Molecular testing, Fine needle aspiration

## Abstract

**Background:**

The preoperative diagnosis of cytologically indeterminate thyroid nodules (ITNs) is very challenging. In this study, we aim to provide an integrated risk assessment for thyroid nodules with indeterminate cytology to guide surgical decision-making, which includes results of blood tests, molecular tests, and repeat fine-needle aspiration biopsy (FNAB).

**Methods:**

The study retrospectively included 265 ITNs between June 2019 and April 2022. According to our integrated risk assessment process that starts with blood testing, followed by supplementary DNA mutation detection on the first FNAB, and finally repeat FNAB, we divided the ITNs into high-risk and low-risk groups. Performance was evaluated with sensitivity, specificity, positive predictive value (PPV), negative predictive value (NPV), area under the receiver operating characteristic curve (AUC), and the consistency between the risk evaluation and histological results.

**Results:**

Of the 265 ITNs, 87 were included in the risk assessment process. The risk assessment had a sensitivity of 84.1%, specificity of 83.3%, PPV of 95.1%, NPV of 57.7%, and AUC of 0.837. The nodules with consistent results between the risk groups and histological outcomes, which included malignant cases in the high-risk group and benign cases in the low-risk group, accounted for 83.9% of all risk-assessed nodules.

**Conclusions:**

These data suggest that the integrated risk assessment might provide proper information for surgical decision-making in patients with ITNs.

**Supplementary Information:**

The online version contains supplementary material available at 10.1186/s12957-023-02917-x.

## Introduction

According to the latest global cancer data released by the American Cancer Society, thyroid cancer has one of the highest incidences of cancer worldwide, ranking seventh among women [1]. Ultrasound is the most commonly used imaging method to assess malignant thyroid nodules based on the Thyroid Imaging Reporting and Data System (TI-RADS) guidelines [2–4]. For thyroid nodules that are difficult to identify only by ultrasound, ultrasound-guided fine-needle aspiration biopsy (FNAB) is usually recommended for routine diagnosis. Currently, FNAB results are divided into six diagnostic categories according to The Bethesda System for Reporting Thyroid Cytopathology (TBSRTC): (I) nondiagnostic or unsatisfactory, (II) benign, (III) atypia of undetermined significance or follicular lesion of undetermined significance (AUS/FLUS), (IV) follicular neoplasm or suspicious for a follicular neoplasm (FN/SFN), (V) suspicious for malignancy (SUSP), and (VI) malignant [5, 6].

Approximately 20% of FNAB of thyroid nodules are classified as Bethesda III/IV/V, which are defined as cytologically indeterminate thyroid nodules (ITNs) [6–8]. ITNs should be treated by surgery or follow-up, depending on the clinical risk factors, ultrasound patterns, and patient preferences [9]. Currently, histopathological examination is the gold standard for diagnosing nodules, and the risk of malignancy in ITNs varies from 5 to 30% [10–14]. In other words, many patients may have undergone unnecessary surgeries. Therefore, the challenge lies in distinguishing between benign and malignant ITNs before surgery [15].

Some research indicates that a single test can help in the management of ITNs. Blood tests for carcinoembryonic antigen (CEA) and calcitonin are helpful for distinguishing medullary thyroid cancer (MTC) from other types of thyroid cancer in a sensitive manner [16, 17]. In addition, the usefulness of CEA and calcitonin was also demonstrated by immunohistochemistry in histopathology [16, 18]. Considering the risk of malignancy of ITNs, the side effects on patients with advanced or metastatic MTC, and the financial burdens of additional imaging evaluation, the use of serum indicators is economical and necessary [19–22]. For ITNs, recent studies have shown that as an alternative to surveillance and diagnostic surgery, molecular testing may be used to supplement the risk assessment of malignant tumors. Two kinds of genetic tests, the Afirma Gene Expression Classifier and ThyroSeq genomic classifier, are the most common products in developed countries [23–25]. Although these products can detect approximately 90% of benign thyroid nodules and can help half of patients with indeterminate cytology avoid diagnostic surgery, they are not yet available in most countries and are expensive. More importantly, these assays are invasive and require at least two additional needle insertions for the analysis of genomic expression. As an alternative, 5 of the most common thyroid cancer-associated somatic mutations (*BRAF*, *HRAS*, *NRAS*, *KRAS*, and *TERT*) have been applied in clinical practice. The detection of these five DNA mutations does not require additional aspiration, and the cytological samples obtained from the first FNAB can be used. The five most common somatic mutations have been widely validated to identify a large proportion of thyroid cancers [26–28]. In addition, it has been reported that repeat aspiration can help to confirm the benign or malignant of ITNs [29–32].

However, combinations of current auxiliary diagnostic methods remain lacking. The aim of our research is to provide a risk assessment tool by integrating serum CEA and calcitonin levels, molecular alterations, and repeat cytology results that might be useful in surgical decision-making. To minimize damage and cost, the risk assessment starts with blood testing, followed by supplementary DNA mutation detection on the first FNAB, and finally repeat FNAB. We describe the results of a retrospective, single-center study validating this evaluation tool in patients with ITNs.

## Methods

### Study population

Our 34-month retrospective study included 254 patients and 265 ITNs at Beijing Hospital between June 2019 and April 2022 (Additional Table 1). Patients were selected on the basis of the following criteria: (a) thyroid nodules are classified as TI-RADS 4 or 5; (b) thyroid nodules are larger than or equal to 1 cm, or less than 1 cm but highly suspected of malignancy based on the doctors’ experience; (c) diagnosis as Bethesda III or Bethesda IV or Bethesda V based on TBSRTC; (d) older than 18 years old and younger than 85 years old. Patients who had history of other cancers, who had previously received radiotherapy in the head and neck region, or had a family history of thyroid cancer were excluded from the study. When available, patient demographics, TI-RADS and Bethesda classifications, DNA molecular alterations, repeat FNAB, and histopathological diagnosis were collected.

This study was approved by the Research Ethics Board of the Beijing Hospital, National Health Commission and performed in accordance with the Declaration of Helsinki (IRB number in Ethical approval: 2021BJYYEC-044-03). All participants provided written informed consent before participation.

### Serum CEA and serum calcitonin measurement

We collected data for serum CEA and serum calcitonin levels from the medical record system of Beijing Hospital. The normal values of CEA and calcitonin were defined as less than 5.0 ng/ml and 19 pg/ml, respectively.

### Fine needle aspiration biopsy

Fine needle aspiration was performed under the guidance of ultrasound by experienced doctors at Beijing Hospital based on Chinese guidelines on the diagnosis and treatment of thyroid nodules and differentiated thyroid carcinomas. According to TBSRTC, the FNAB results were classified into six diagnostic categories and confirmed by two experienced pathologists. If the specimen meets the criteria, mutation testing was recommended. Gene mutations were detected on the first fine-needle aspiration samples by amplification refractory mutation system, including *BRAF* exon 15, *HRAS* exon 3, *NRAS* exon 2/3/4, *KRAS* exon 2/3/4, *TERT* promoter mutations (C228T and C250T).

### Surgical procedure and histological examination

Total thyroidectomy or lobectomy was performed by one expert surgeon in Beijing Hospital depending on the clinical risk factors, ultrasound patterns, FNAB, and patients’ preferences. With the help of an ultrasonic diagram, the surgeon located the nodule and oriented the resected specimen for pathological diagnosis. The results of a blinded histopathological examination were used as the reference standard.

### Statistical analysis

Statistical analysis was conducted using SPSS software, version 26. Student’s *t* tests and Mann–Whitney *U*-rank sum tests were used to analyze continuous variables. Chi-square test was used to analyze categorical variables. Based on established methods, sensitivity, specificity, and positive and negative predictive values were calculated [33]. *P* values less than 0.05 were considered statistically significant.

## Results

### Patient and nodule characteristics

**Fig. 1:**
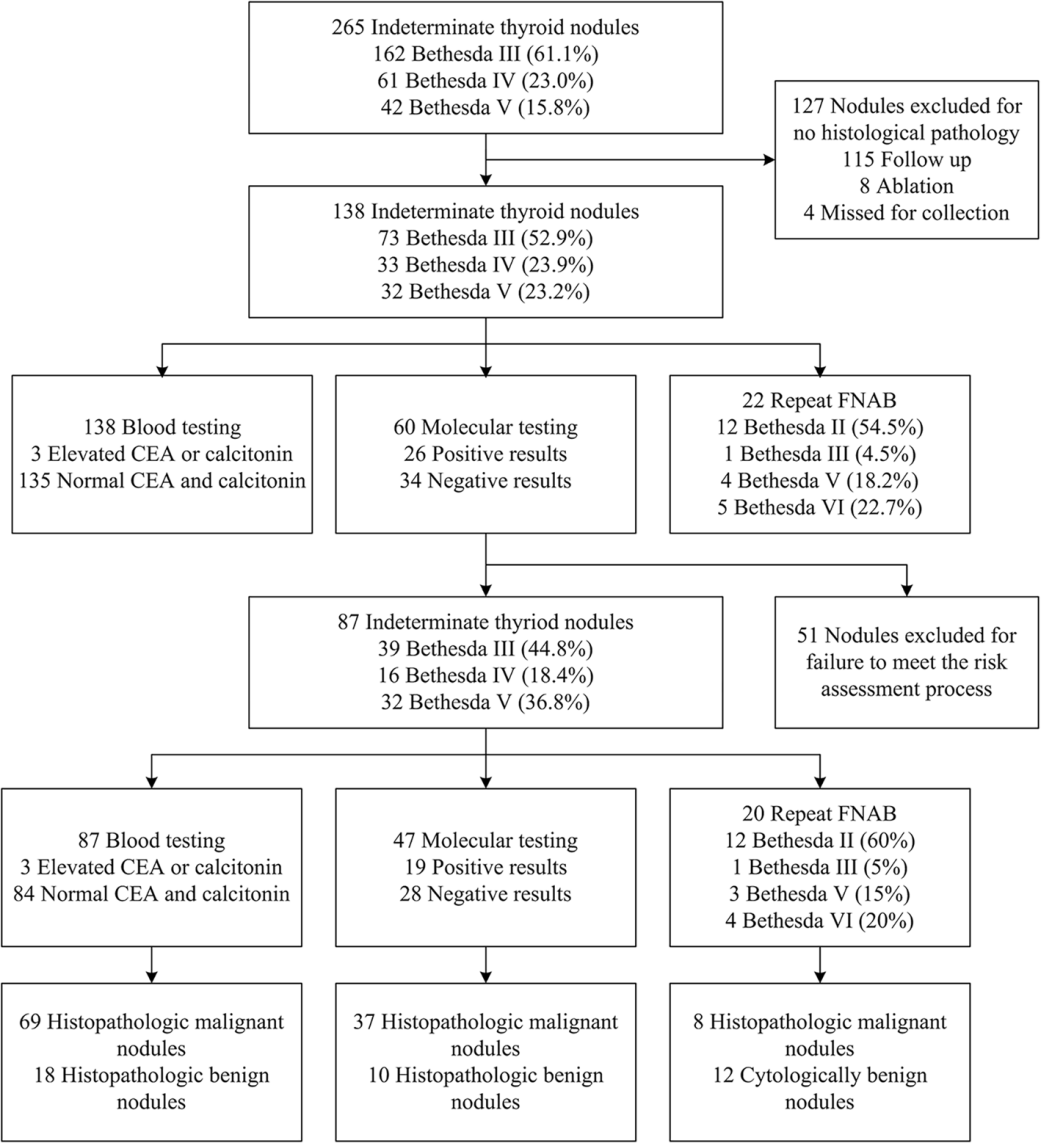
Flowchart for the cytologically indeterminate thyroid nodules included in risk assessment

To evaluate the clinical characteristics of ITNs, we retrospectively collected the data of 265 ITNs from 254 patients at Beijing Hospital in China over a 34-month period. Of the 265 nodules, 61.1%, 23%, and 15.8% of the nodules were classified as Bethesda III, Bethesda IV, and Bethesda V, respectively (Fig. 1). There were more females than males, with the proportion of females being 79.1%. For 115 nodules with uncertain cytology, molecular tests were performed. Additional Table 1 shows the baseline features of the patients and nodules.

We finally enrolled 138 nodules with histological diagnosis (Additional Table 2). Of the excluded samples, 115 were followed up regularly (81 cases were Bethesda III, 24 cases were Bethesda IV, 10 cases were Bethesda V), 8 patients chose ablation instead of an operation (5 cases were Bethesda III, 3 cases were Bethesda IV), and 4 cases were treated surgically in other hospitals. All 127 nodules were excluded from the final analysis (Fig. 1).

### Prevalence rate of malignant tumors among ITNs

A total of 52.9% of 138 samples were considered Bethesda III, 23.9% were Bethesda IV, and 23.2% were Bethesda V. The malignancy rates among all indeterminate nodules were 75.4%, 78.1%, 51.5%, and 93.8% in Bethesda III, IV, and V lesions, respectively. No significant differences were found in age, sex, or nodule size between the malignant (104 samples) and benign groups (34 samples). Additional Table 3–6 show histology and molecular alterations of ITNs. In 2 cases of follicular thyroid carcinomas with molecular changes, 1 patient carried both *HRAS* and *TERT* mutations, and 1 patient carried *TERT* mutation. *NRAS* mutation was found in 1 sample, which was a noninvasive follicular thyroid neoplasm with papillary-like nuclear features (NIFTP).

### Integrated assessment can identify high-risk ITNs

**Fig. 2:**
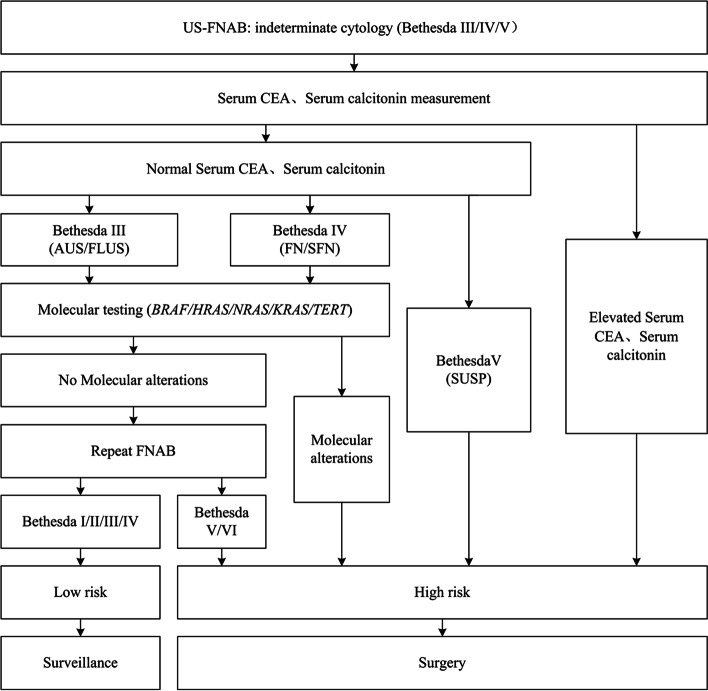
Risk assessment for cytologically indeterminate thyroid nodules with integrated serum carcinoembryonic antigen and calcitonin measurement, molecular testing, and repeated aspiration

According to our integrated risk assessment, we divided the ITNs into high-risk and low-risk groups (Fig. 2). Serum CEA and serum calcitonin levels were measured in all ITNs patients, among which 3 patients showed elevated serum CEA and calcitonin. They were classified as high risk and finally diagnosed with medullary thyroid carcinoma (2 cases were Bethesda IV, and 1 case was Bethesda V). In samples with normal serum CEA and serum calcitonin levels, 32 Bethesda V nodules were classified as high risk, and others were detected by molecular testing, including for *BRAF*, *HRAS*, *NRAS*, *KRAS*, and *TERT*. We classified those with molecular alterations as high risk and a portion of the other patients undergo repeat FNAB at least 1 month after the first aspiration. We considered nodules classified as Bethesda V or Bethesda VI by repeat biopsy to be high risk, while others were classified as low risk.

Among 265 cases of indeterminate fine-needle aspirates, 87 cases were included in the risk assessment (Tables 1, 2, 3, and 4). The risk assessment correctly identified 58 out of the 69 malignant samples as high risk and 15 out of 18 nonmalignant samples as low risk, resulting in a sensitivity of 84.1%, specificity of 83.3%, positive predictive value (PPV) of 95.1%, negative predictive value (NPV) of 57.7%, and area under the receiver operating characteristic curve (AUC) of 0.837. To compare the results of the risk evaluation with histopathology, we defined the malignant cases in the high-risk group and the benign cases in the low-risk group as the consistent results group and the malignant cases in the low-risk group and the benign cases in the high-risk group as the inconsistent results group. The findings indicated that the coincidence rates of the assessed cohort and the excluded cohort were 83.9% and 31.4%, respectively. For nodules classified as Bethesda III, the sensitivity was 75%, specificity was 90%, PPV was 95.5%, NPV was 58.8%, and AUC was 0.83. For nodules classified as Bethesda IV, the sensitivity was 63.6%, specificity was 100%, PPV was 100%, NPV was 55.6%, and AUC was 0.818.

**Table 1:** Performance of the risk assessment tool for cytologically indeterminate samples. Performance on cytologically indeterminate thyroid nodules (*N* = 87)

Risk assessment	Malignant reference standard	Benign reference standard
High risk	58	3
Low risk	11	15

Sensitivity, 84.1%; specificity, 83.3%; PPV, 95.1%; NPV, 57.7%; AUC, 0.837; *p* < 0.05

*Abbreviations*: *AUS/FLUS* atypia (or follicular lesions) of undetermined significance, *FN/SFN* follicular neoplasms or lesions suspicious for follicular neoplasm, *SUSP* suspicious of malignancy, *NPV* negative predictive value, *PPV* positive predictive value, *AUC* the area under receiver operating characteristic curve

**Table 2:** Performance of the risk assessment tool for cytologically indeterminate samples. Consistency of risk evaluation and histological results (*N* = 138)

	Consistency	Inconsistency
Risk assessed	73 (83.9%)	14
No risk assessed	16 (31.4%)	35

*p* < 0.05

*Abbreviations*: *AUS/FLUS* atypia (or follicular lesions) of undetermined significance, *FN/SFN* follicular neoplasms or lesions suspicious for follicular neoplasm, *SUSP* suspicious of malignancy, *NPV* negative predictive value, *PPV* positive predictive value, *AUC* the area under receiver operating characteristic curve

**Table 3:** Performance of the risk assessment tool for cytologically indeterminate samples. Performance of integrated risk assessment on AUS/FLUS and FN/SFN (*N* = 55)

Risk assessment	Malignant reference standard	Benign reference standard
High risk	28	1
Low risk	11	15

Sensitivity, 71.8%; specificity, 93.8%; PPV, 96.6%; NPV, 57.7%; AUC, 0.828; *p* < 0.05

*Abbreviations*: *AUS/FLUS* atypia (or follicular lesions) of undetermined significance, *FN/SFN* follicular neoplasms or lesions suspicious for follicular neoplasm, *SUSP* suspicious of malignancy, *NPV* negative predictive value, *PPV* positive predictive value, *AUC* the area under receiver operating characteristic curve

**Table 4:** Performance of the risk assessment tool for cytologically indeterminate samples. Performance of repeat aspiration when no molecular alterations detected on AUS/FLUS and FN/SFN (*N* = 13)

Risk assessment	Malignant reference standard	Benign reference standard
High risk	6	0
Low risk	1	6

Sensitivity, 85.7%; specificity, 100%; PPV, 100%; NPV, 85.7%; AUC, 0.929; *p* < 0.05

*Abbreviations*: *AUS/FLUS* atypia (or follicular lesions) of undetermined significance, *FN/SFN* follicular neoplasms or lesions suspicious for follicular neoplasm, *SUSP* suspicious of malignancy, *NPV* negative predictive value, *PPV* positive predictive value, *AUC* the area under receiver operating characteristic curve

With the use of repeat aspiration, 7 of 8 the papillary thyroid cancer can be identified as high-risk (Bethesda V or VI), and 12 of the 20 ITNs were reclassified as Bethesda II. With the use of repeat aspiration after first FNAB diagnosed as Bethesda III or IV and with no molecular alterations detected, 6 of 7 papillary thyroid cancer could be identified as high-risk (Bethesda V or VI), and 6 of 13 ITNs were reclassified as Bethesda II (Additional Table 7).

In the assessment system, 3 patients were found to have false-positive results (Additional Table 8). One was a 30-year-old female, classified as Bethesda III, and she carried the *BRAF*^V600E^ mutation. The nodule was located on the right side of the thyroid gland, with a size of 0.4 cm. The other two were a 36-year-old female and a 55-year-old male. Their left nodules were classified as Bethesda V with *BRAF*^V600E^ mutations and sizes of 0.3 cm, and histology showed no cancer in one case and a nodular goiter in the other case, while their right nodules were diagnosed as papillary thyroid carcinomas. Fifty-one nodules were excluded from the risk assessment. Additional Table 9 shows histology of nodules excluded from the risk assessment.

## Discussion

We provided a risk assessment method for patients with indeterminate cytology diagnoses based on the integration of blood test results, molecular signatures, and repeat cytological findings of thyroid nodules. It is critical to diagnose MTC at an early stage due to the relatively high incidence of metastasis [20, 22]. The levels of serum CEA and calcitonin have been proven to be risk indicators for medullary thyroid cancer and are essential to guide appropriate surgical treatment [34, 35]. Nowadays, screening for CEA and calcitonin has already become a routine test for high-risk thyroid nodules in tertiary care hospitals in China, and the cost is covered by the National Health Insurance. In our study, blood tests identified all 3 medullary thyroid cancers of 87 ITNs, which is consistent with previous research by others. Some studies have shown that a single method, such as molecular testing or repeat FNAB, can help determine the prevalence of malignancy in ITNs [7, 36–46]. In recent years, two kinds of gene tests have appeared on the market, the Afirma Gene Expression Classifier and ThyroSeq genomic classifier. A randomized clinical trial including 346 patients with indeterminate nodules indicated that both products have high specificity and can help 49% of patients with ITNs avoid diagnostic surgery [23]. In this case series, as these molecular tests mentioned above are not yet available in China and are expensive, the department of pathology detected the most common cancer-associated somatic mutations (*BRAF*, *HRAS*, *NRAS*, *KRAS*, and *TERT*) in 115 of 265 specimens from ITNs. These 5 molecular alterations are frequently represented in malignant thyroid nodules, especially *BRAF.* Twenty-two of 24 ITNs carrying the *BRAF*^V600E^ mutation were diagnosed as papillary thyroid cancer. As reported in the literature, *BRAF* mutations have high specificity, and *RAS* mutations have more limits for relatively low specificity [47–51]. Our findings also showed that almost all detected mutations were *BRAF* mutations, but future studies will be needed to verify the presence of these mutations in a larger sample size. Our data showed that molecular detection might be beneficial to thyroid nodules because evaluations of the genetic alterations of Bethesda III and Bethesda IV samples improved the specificity of a cancer diagnosis to 90.0%. Considering the possibility of false negatives in molecular tests, 13 patients with no molecular alterations underwent repeat FNAB during follow-up according to the suspected ultrasound characteristics and patients’ wishes, and the second biopsies were performed at least 1 month after the first ones (Additional Table 7). Our research also indicated that repeat aspiration is related to improved specificity in the diagnosis of ITNs with high-risk features.

A possible strength of our study is that we integrated the results of three of the most frequently used tests for ITNs into a surgical decision-oriented tool. Considering the Afirma Gene Expression Classifier and ThyroSeq genomic classifier are not yet available in China and are expensive, 5 of the most common cancer-associated somatic mutations (*BRAF, HRAS, NRAS, KRAS* and *TERT*) were offered in our hospital. Because the results of molecular testing could be false negatives, some patients underwent repeated biopsies during follow-up. We developed the risk assessment process based on the principle of minimizing costs and minimizing damage. The evaluation tool starts with blood test results (CEA and calcitonin), followed by molecular tests, and finally repeat FNAB results. Our study finally retrospectively collected 87 ITNs and the assessment method was validated on more than 10 kinds of histopathological types. All the ITNs were highly suspected of malignancy on ultrasound scans and cytologically diagnosed as Bethesda III or Bethesda IV or Bethesda V based on TBSRTC. By using the risk evaluation tool, we could accurately identify the patients with high-risk ITNs that might benefit from surgical treatment. The results showed that the specificity was 90.0% for Bethesda III lesions and 100% for Bethesda IV lesions, indicating that malignant ITNs with these cytologic findings can be correctly distinguished from benign ITNs. In addition, 73 out of 87 histological malignant ITNs were correctly distinguished, showing that the integrated assessment tool can help to improve the accuracy of the preoperative diagnosis of ITNs. To be more specific, 58 (84.1%) malignant nodules will be missed without using the risk tool, and 15 (83.3%) benign nodules will be submitted to surgery [52, 53]. We compared the diagnostic performance of our risk assessment with different tests (Additional Table 10). It showed that this integrated tool was not inferior to expensive multigene products. The risk assessment is a beneficial and cost-effective method for clarifying the malignant or benign nature of ITNs, taking into account the side effect of extensive surgery in patients with metastatic thyroid cancer and the cost of active surveillance [52–57].

However, the lack of some equivalent risk consideration is a weakness of this integrated assessment. Given that germline mutation plays an important role in the evaluation of thyroid nodules and is detected on peripheral blood samples, it could be added in the subsequent evaluation, especially for patients with a family history [58, 59]. Besides, NPV and PPV depend on the prevalence rate of disease in the population, and our research showed that the prevalence rate of cancer in ITNs was as high as 70.1% in our 87 samples. For ITNs classified as Bethesda III or IV or V, the risk of malignancy was 78.1%, 51.5%, and 93.8%, respectively. One factor for the high prevalence rate of malignancy is that patients who chose surgery had malignant signs of malignancy and surgeons recommended surgery based on their experience. Of the 265 nodules initially collected, 127 did not choose immediate surgery. Twenty out of 104 nodules that had histological results carried at least one of the most common cancer-associated molecular alterations and 21 patients undergone repeat FNAB. Another explanation is that the rate of malignancy of ITNs in China may be underestimated. A study that recruited 140 samples in China showed that the malignant rate was 74.1% for resected ITNs [60]. Another multicenter study in Israel included 810 patients and indicated that the malignancy rates were higher than those reported earlier. The authors suggested that doctors should use validated data for their own country in addition to published values [61].

It should be observed that the NPV is not very high, and it is the most meaningful value to avoid surgery in patients with negative results. Although the risk-assessment tool could prevent 15 out of 18 unnecessary surgeries, it also incorrectly stopped 11 out of 69 surgeries of malignant nodules. Given that 7 false-negative nodules in this study were histologically diagnosed as papillary thyroid microcarcinoma and 3 were noninvasive follicular thyroid neoplasm with papillary-like nuclear features, we recommend that active surveillance can be adopted as a treatment option for the low-risk ITNs as there are characteristic features of indolence of these subtypes, and either tumor enlargement or the novel appearance of nodal or distant metastasis is the indication for surgery. Besides, multiple studies have shown that delayed surgery is safe [62–64]. It is important to note that NPV changes accordantly with the risk of malignancy in different Bethesda classifications, which indicates that the risk assessment is more critical for ITNs with lower risk of malignancy. Moreover, the three nodules with false-positive results cannot be ignored. As one was a Bethesda III nodule with a *BRAF*^V600E^ mutation detected, the two others were Bethesda V nodules, and all of the nodules were less than 0.5 cm, we believe that it is possible that postoperative histological pathology missed the neoplastic foci. It is worth mentioning that the small sample size and single-center nature of this research limit the generalizability of the results; therefore, more prospective studies are needed to verify the efficacy of this risk assessment method.

It is also worth noting that one limitation of this study is the small sample size. In our study, 265 ITNs were included in the beginning, but the risk assessment was done on only 87 samples, of which only 47 (54%) underwent genetic testing. This finding suggests that this risk assessment tool needs to be evaluated prospectively in larger sample populations in future studies.

## Conclusions

This study proposed a risk assessment tool by integrating serum CEA and calcitonin measurements, molecular testing, and repeat aspiration that seemed to provide a useful evaluation method to help surgical decision-making for patients with ITNs. This risk assessment can bring benefits to surgeons and patients when considering surgical treatment, and we hope that this study will provide supporting information for clinical recommendations and give surgeons more confidence in making decisions for their patients.

## Supplementary Information

**Additional file 1: Table S1.** Baseline demographic and clinical characteristics of patients with indeterminate thyroid nodules. **Table S2.** Baseline demographic and clinical characteristics of indeterminate thyroid nodules with histology (*N*=138). **Table S3.** Histology and molecular alterations of indeterminate thyroid nodules (*N*=138). **Table S4.** Histology and molecular alterations of Atypia (or Follicular Lesions) of Undetermined Significance Thyroid Nodules (*N*=73). **Table S5.** Histology and molecular alterations of Follicular Neoplasms or Lesions Suspicious for Follicular Neoplasm Thyroid Nodules (*N*=33). **Table S6.** Histology and molecular alterations of Suspicious of Malignancy Thyroid Nodules (*N*=32). **Table S7.** Repetitive and final histological diagnosis of ITNs included in the risk assessment (*N*=20). **Table S8.** Cytologic findings and histopathological diagnosis in 3 patients with false-positive results. **Table S9.** Histology of indeterminate thyroid nodules excluded for the risk assessment (*N*=51). **Table S10.** Performance of different risk assessment tools for cytologically indeterminate thyroid nodules. **Table S11.** Molecular alterations and histologic diagnosis of ITNs included in the risk assessment (*N*=47).

## Data Availability

The datasets used and/or analyzed during the current study are available from the corresponding author on reasonable request. The datasets supporting the conclusions of this article are included within the article (and its additional files).

## Abbreviations

TI-RADS: Thyroid Imaging Reporting and Data System
FNAB: Fine-needle aspiration biopsy
TBSRTC: The Bethesda System for Reporting Thyroid Cytopathology
AUS/FLUS: Atypia of undetermined significance or follicular lesion of undetermined significance
FN/SFN: Follicular neoplasm or suspicious for a follicular neoplasm
SUSP: Suspicious for malignancy
ITNs: Indeterminate thyroid nodule
CEA: Carcinoembryonic antigen
NIFTP: Noninvasive follicular thyroid neoplasm with papillary-like nuclear features
PPV: Positive predictive value
NPV: Negative predictive value
AUC: Area under the receiver operating characteristic curve
