# Supplementary material for: Risk assessment of cytologically indeterminate thyroid nodules with integrated molecular testing and repeat biopsy: a surgical decision-oriented tool
Source: World J Surg Oncol. 2023 Feb 3;21:34. doi: 10.1186/s12957-023-02917-x (PMC9896714; doi:10.1186/s12957-023-02917-x)
Supplement: Supplementary file 1 — Additional file 1: Table S1. Baseline demographic and clinical characteristics of patients with indeterminate thyroid nodules. Table S2. Baseline demographic and clinical characteristics of indeterminate thyroid nodules with histology (N=138). Table S3. Histology and molecular alterations of indeterminate thyroid nodules (N=138). Table S4. Histology and molecular alterations of Atypia (or Follicular Lesions) of Undetermined Significance Thyroid Nodules (N=73). Table S5. Histology and molecular alterations of Follicular Neoplasms or Lesions Suspicious for Follicular Neoplasm Thyroid Nodules (N=33). Table S6. Histology and molecular alterations of Suspicious of Malignancy Thyroid Nodules (N=32). Table S7. Repetitive and final histological diagnosis of ITNs included in the risk assessment (N=20). Table S8. Cytologic findings and histopathological diagnosis in 3 patients with false-positive results. Table S9. Histology of indeterminate thyroid nodules excluded for the risk assessment (N=51). Table S10. Performance of different risk assessment tools for cytologically indeterminate thyroid nodules. Table S11. Molecular alterations and histologic diagnosis of ITNs included in the risk assessment (N=47). [file 12957_2023_2917_MOESM1_ESM.docx]

**Additional Table 1** Baseline demographic and clinical characteristics of patients with indeterminate thyroid nodules

|  | Indeterminate Thyroid Nodules |
| --- | --- |
| Total no. |  |
| Nodules | 265 |
| Patients | 254 |
| Age of patients-yr |  |
| Mean | 48 (±12.698) |
| Range | 21-81 |
| Sex-no. of patients (%) |  |
| Male | 53 (20.9) |
| Female | 201 (79.1) |
| Size on ultrasonography-cm |  |
| Median | 0.70 |
| Range | 0.30-5.30 |
| Location-no. of nodules (%) |  |
| Left | 97 (36.6) |
| Right | 131 (49.4) |
| Isthmus | 7 (2.6) |
| TBSRTC -no. of nodules (%) |  |
| III (AUS/FLUS) | 162 (61.1) |
| IV (FN/SFN) | 61 (23) |
| V (SUSP) | 42 (15.8) |

Abbreviations: TBSRTC, Bethesda System for Reporting Thyroid Cytopathology

**Additional Table 2** Baseline demographic and clinical characteristics of indeterminate thyroid nodules with histology (N=138)

|  | Malignant | Benign | *p* value |
| --- | --- | --- | --- |
| Total no. | 104 (75.4%) | 34 (24.6%) |  |
| Age of patients-yr |  |  |  |
| Mean | 46.79 (±12.185) | 49.29 (±12.674) | 0.305 |
| Range | 24-76 | 25-69 |  |
| Sex-no. of patients (%) |  |  |  |
| Male | 17 (65.4%) | 9 (34.6%) | 0.190 |
| Female | 87 (77.7%) | 25 (22.3%) |  |
| Size on ultrasonography-cm |  |  |  |
| Median | 0.60 | 1.05 | **0.010** |
| Range | 0.30-4.20 | 0.30-4.90 |  |
| TI-RADS (%) |  |  |  |
| 3 | 7 (50%) | 7 (50%) | **0.029** |
| 4 | 76 (76.8%) | 23 (23.2%) |  |
| 5 | 6 (100%) | 0 |  |
| 6 | 6 (100%) | 0 |  |
| TBSRTC - no. of nodules (%) |  |  |  |
| III (AUS/FLUS) | 57 (78.1%) | 16 (21.9%) | **＜0.05** |
| IV (FN/SFN) | 17 (51.5%) | 16 (48.5%) |  |
| V (SUSP) | 30 (93.8%) | 2 (6.3%) |  |
| *BRAF*^V600E^ mutation |  |  |  |
| Positive | 22 (91.7%) | 2 (8.3%) | 0.109 |
| Negative | 27 (75%) | 9 (25%) |  |

Values in bold are statistically significant

Abbreviations: TI-RADS, Thyroid Imaging Reporting and Data System; TBSRTC, Bethesda System for Reporting Thyroid Cytopathology

**Additional Table 3** Histology and molecular alterations of indeterminate thyroid nodules (N=138)

| TBSRTC | Histology | Malignant | Benign | *BRAF^V600E^*  Mutation | *TERT*  Mutation | *HRAS*  Mutation | *NRAS*  Mutation |
| --- | --- | --- | --- | --- | --- | --- | --- |
| Indeterminate Thyroid  Nodules | PTC | 89 (64.5%) |  | 22 |  |  |  |
|  | Classic variant | 53 |  |  |  |  |  |
|  | Follicular variant | 29 |  |  |  |  |  |
|  | FTC | 2 (1.4%) |  |  | 2 | 1 |  |
|  | MTC | 3 (2.2%) |  |  |  |  |  |
|  | Hürthle-cell carcinoma | 1 (0.7%) |  |  |  |  |  |
|  | NIFTP | 9 (6.5%) |  |  |  |  | 1 |
|  | Found no cancer |  | 2 (1.4%) | 1 |  |  |  |
|  | Nodular goiter |  | 6 (4.3%) |  |  |  |  |
|  | Follicular adenoma |  | 7 (5.1%) |  |  |  |  |
|  | Hashimoto's thyroiditis |  | 7 (5.1%) | 1 |  |  |  |
|  | Benign follicular nodule |  | 12 (8.7%) |  |  |  |  |

Abbreviations: TBSRTC, Bethesda System for Reporting Thyroid Cytopathology; PTC, Papillary thyroid carcinoma; FTC, Follicular thyroid carcinoma; MTC, Medullary thyroid cancer; NIFTP, Noninvasive follicular thyroid neoplasm with papillary-like nuclear features

**Additional Table 4** Histology and molecular alterations of Atypia (or Follicular Lesions) of Undetermined Significance Thyroid Nodules (N=73)

| TBSRTC | Histology | Malignant | Benign | *BRAF^V600E^*  Mutation | *TERT*  Mutation | *HRAS*  Mutation | *NRAS*  Mutation |
| --- | --- | --- | --- | --- | --- | --- | --- |
| AUS/FLUS | PTC | 54 (74.0%) |  | 15 |  |  |  |
|  | Classic variant | 32 |  |  |  |  |  |
|  | Follicular variant | 17 |  |  |  |  |  |
|  | FTC | 0 |  |  |  |  |  |
|  | MTC | 0 |  |  |  |  |  |
|  | Hürthle-cell carcinoma | 0 |  |  |  |  |  |
|  | NIFTP | 3 (4.1%) |  |  |  |  |  |
|  | Found no cancer |  | 1 (1.4%) | 1 |  |  |  |
|  | Nodular goiter |  | 3 (4.1%) |  |  |  |  |
|  | Follicular adenoma |  | 0 |  |  |  |  |
|  | Hashimoto's thyroiditis |  | 3 (4.1%) |  |  |  |  |
|  | Benign follicular nodule |  | 9 (12.3%) |  |  |  |  |

Abbreviations: TBSRTC, Bethesda System for Reporting Thyroid Cytopathology; PTC, Papillary thyroid carcinoma; FTC, Follicular thyroid carcinoma; MTC, Medullary thyroid cancer; NIFTP, Noninvasive follicular thyroid neoplasm with papillary-like nuclear features; AUS/FLUS, Atypia (or Follicular Lesions) of Undetermined Significance

**Additional Table 5** Histology and molecular alterations of Follicular Neoplasms or Lesions Suspicious for Follicular Neoplasm Thyroid Nodules (N=33)

| TBSRTC | Histology | Malignant | Benign | *BRAF^V600E^*  Mutation | *TERT*  Mutation | *HRAS*  Mutation | *NRAS*  Mutation |
| --- | --- | --- | --- | --- | --- | --- | --- |
| FN/SFN | PTC | 7 (21.2%) |  | 2 |  |  |  |
|  | Classic variant | 5 |  |  |  |  |  |
|  | Follicular variant | 2 |  |  |  |  |  |
|  | FTC | 1 (3.0%) |  |  | 1 | 1 |  |
|  | MTC | 2 (6.1%) |  |  |  |  |  |
|  | Hürthle-cell carcinoma | 1 (3.0%) |  |  |  |  |  |
|  | NIFTP | 6 (18.2%) |  |  |  |  | 1 |
|  | Found no cancer |  | 0 |  |  |  |  |
|  | Nodular goiter |  | 2 (6.1%) |  |  |  |  |
|  | Follicular adenoma |  | 7 (21.2%) |  |  |  |  |
|  | Hashimoto's thyroiditis |  | 4 (12.1%) |  |  |  |  |
|  | Benign follicular nodule |  | 3 (9.1%) |  |  |  |  |

Abbreviations: TBSRTC, Bethesda System for Reporting Thyroid Cytopathology; PTC, Papillary thyroid carcinoma; FTC, Follicular thyroid carcinoma; MTC, Medullary thyroid cancer; NIFTP, Noninvasive follicular thyroid neoplasm with papillary-like nuclear features; FN/SFN, Follicular Neoplasms or Lesions Suspicious for Follicular Neoplasm

**Additional Table 6** Histology and molecular alterations of Suspicious of Malignancy Thyroid Nodules (N=32)

| TBSRTC | Histology | Malignant | Benign | *BRAF^V600E^*  Mutation | *TERT*  Mutation | *HRAS*  Mutation | *NRAS*  Mutation |
| --- | --- | --- | --- | --- | --- | --- | --- |
| SUSP | PTC | 28 (87.5%) |  | 5 |  |  |  |
|  | Classic variant | 16 |  |  |  |  |  |
|  | Follicular variant | 10 |  |  |  |  |  |
|  | FTC | 1 (3.1%) |  |  | 1 |  |  |
|  | MTC | 1 (3.1%) |  |  |  |  |  |
|  | Hürthle-cell carcinoma | 0 |  |  |  |  |  |
|  | NIFTP | 0 |  |  |  |  |  |
|  | Found no cancer |  | 1 (3.1%) | 1 |  |  |  |
|  | Nodular goiter |  | 1 (3.1%) |  |  |  |  |
|  | Follicular adenoma |  | 0 |  |  |  |  |
|  | Hashimoto's thyroiditis |  | 0 |  |  |  |  |
|  | Benign follicular nodule |  | 0 |  |  |  |  |

Abbreviations: TBSRTC, Bethesda System for Reporting Thyroid Cytopathology; PTC, Papillary thyroid carcinoma; FTC, Follicular thyroid carcinoma; MTC, Medullary thyroid cancer; NIFTP, Noninvasive follicular thyroid neoplasm with papillary-like nuclear features; SUSP, Suspicious of Malignancy

**Additional Table 7** Repetitive and final histological diagnosis of ITNs included in the risk assessment (N=20)

| Case ID | First FNAB  diagnosis | Molecular alterations | Second FNAB diagnosis | Histologic  diagnosis |
| --- | --- | --- | --- | --- |
| 1 | III | Not detected | VI | PTC |
| 2 | III | Not detected | VI | PTC |
| 3 | III | Not detected | V | PTC |
| 4 | III | Not detected | V | PTC |
| 5 | III | Not detected | VI | PTC |
| 6 | III | Not detected | III | PTC |
| 7 | III | Not detected | II | N/A |
| 8 | III | Not detected | II | N/A |
| 9 | III | Not detected | II | N/A |
| 10 | III | Not detected | II | N/A |
| 11 | III | Not detected | II | N/A |
| 12 | IV | Not detected | V | PTC |
| 13 | IV | Not detected | II | N/A |
| 14 | III | N/A | VI | PTC |
| 15 | III | N/A | II | N/A |
| 16 | III | N/A | II | N/A |
| 17 | III | N/A | II | N/A |
| 18 | III | N/A | II | N/A |
| 19 | IV | N/A | II | N/A |
| 20 | IV | N/A | II | N/A |

Abbreviations: FNAB, Fine-needle aspiration biopsy; PTC, Papillary thyroid cancer; N/A, Not applicable

**Additional Table 8** Cytologic findings and histopathological diagnosis in 3 patients with false-positive results

| Sex | Age | TBSRTC | *BRAF^V600E^* mutation | Location | Size -cm | TI-RADS | Histopathological Diagnosis | Other |
| --- | --- | --- | --- | --- | --- | --- | --- | --- |
| Female | 30 | III | Positive | Right | 0.4 | N/A | Hashimoto's Thyroiditis | N/A |
| Female | 36 | V | Positive | Left | 0.3 | 4a | No Cancer | PTC of the Right Lobe |
| Male | 55 | V | N/A | Left | 0.3 | 4a | Nodular Goiter | PTC of the Right Lobe |

Abbreviations: TBSRTC, Bethesda System for Reporting Thyroid Cytopathology; TI-RADS, Thyroid Imaging Reporting and Data System; PTC, Papillary thyroid carcinoma; N/A, Not applicable

**Additional Table 9** Histology of indeterminate thyroid nodules excluded for the risk assessment (N=51)

| Category | Number | Percentage (%) |
| --- | --- | --- |
| PTC | 30 | 58.8 |
| Hürthle-cell Adenoma with Active Growth and Focal Visible Envelope Invasion. | 1 | 2.0 |
| NIFTP | 4 | 7.8 |
| Nodular Goiter | 5 | 9.8 |
| Follicular Adenoma | 7 | 13.7 |
| Hashimoto's Thyroiditis | 4 | 7.8 |

Abbreviations: PTC, Papillary thyroid carcinoma; NIFTP, Noninvasive follicular thyroid neoplasm with papillary-like nuclear features

**Additional Table 10** Performance of different risk assessment tools for cytologically indeterminate thyroid nodules

| **Author** | **Country** | **Risk assessment tool** | **TBSRTC** | **No. of surgeries** | **Sensitivity** | **Specificity** | **PPV** | **NPV** | **ROM** |
| --- | --- | --- | --- | --- | --- | --- | --- | --- | --- |
| Present study | China | The integrated risk assessment tool | III/IV/V | 87 | 84.1% | 83.3% | 95.1% | 57.7% | 70.1% |
| Chunfang Hu (2022) [1] | China | Novel RNA panel | III/IV | 58 | 93% | 40% | 81.6% | 66.7% | 74.1% |
| Chunfang Hu (2022) [1] | China | Novel DNA-RNA panel | III/IV | 58 | 88.4% | 53.3% | 84.4% | 61.5% | 74.1% |
| Carla Colombo  (2021) [2] | Italy | The thyroid risk score (TRS) | III/IV | 65 | 72% | 80% | 69% | 82% | 38.5% |
| Erik K. Alexander (2012) [3] | America | Afirma gene expression classifier | III/IV/V | 265 | 92% | 52% | 47% | 93% | 32% |
| Rachel C Jug (2018) [4] | America | Afirma gene expression classifier | III/IV/V | 96 | 100% | 18.3% | 30.1% | 100% | 26% |
| Rachel C Jug (2018) [4] | America | ThyroSeq | III/IV/V | 47 | [4]85.7% | 80% | 42.9% | 97% | 15% |
| Gila Lithwick-Yanai  (2017) [5] | Israel | RosettaGX Reveal | III/IV/V | 189 | 85% | 72% | 59% | 91% | 32.3% |
| Cristina Alina Silaghi  (2021) [6] | 25 studies | Afirma gene expression classifier | III/IV/V | N/A | 97% | 19% | 39% | 91% | N/A |
| Cristina Alina Silaghi  (2021) [6] | 4 studies | Afirma gene sequencing  classifier | III/IV | N/A | 95% | 51% | 60% | 91% | N/A |
| Cristina Alina Silaghi  (2021) [6] | 9 studies | ThyroSeq V2 | III/IV/V | N/A | 86% | 75% | 51% | 95% | N/A |
| Cristina Alina Silaghi  (2021) [6] | 4 studies | ThyroSeq V3 | III/IV/V | N/A | 99% | 64% | 78% | 96% | N/A |

Abbreviations: TBSRTC, Bethesda System for Reporting Thyroid Cytopathology; PPV, Positive predictive value; NPV, Negative predictive value; ROM, Risk of malignancy; N/A, Not applicable.

**Additional Table 11** Molecular alterations and histologic diagnosis of ITNs included in the risk assessment (N=47)

| Sum of cases | FNAB diagnosis | Molecular alterations | Histologic diagnosis |
| --- | --- | --- | --- |
| 15 | III | *BRAF*^V600E^ | PTC |
| 1 | III | *BRAF*^V600E^ | Hashimoto's thyroiditis |
| 11 | III | None | PTC |
| 1 | III | None | NIFTP |
| 1 | III | None | Found no cancer |
| 5 | III | None | N/A |
| 2 | IV | *BRAF*^V600E^ | PTC |
| 1 | IV | *HRAS/TERT* | FTC |
| 2 | IV | None | PTC |
| 1 | IV | None | MTC |
| 1 | IV | None | Hürthle-cell carcinoma |
| 3 | IV | None | NIFTP |
| 2 | IV | None | Hashimoto's thyroiditis |
| 1 | IV | None | N/A |

Abbreviations: FNAB, Fine-needle aspiration biopsy; PTC, Papillary thyroid cancer; FTC, Follicular thyroid carcinoma; MTC, Medullary thyroid cancer; NIFTP, Noninvasive follicular thyroid neoplasm with papillary-like nuclear features; N/A, Not applicable.

Reference

1. Hu C, Jing W, Chang Q, Zhang Z, Liu Z, Cao J, et al. Risk stratification of indeterminate thyroid nodules by novel multigene testing: a study of Asians with a high risk of malignancy. Mol Oncol. 2022;16(8):1680-93.

2. Colombo C, Muzza M, Pogliaghi G, Palazzo S, Vannucchi G, Vicentini L, et al. The thyroid risk score (TRS) for nodules with indeterminate cytology. Endocr Relat Cancer. 2021;28(4):225-35.

3. Wong KS, Angell TE, Strickland KC, Alexander EK, Cibas ES, Krane JF, et al. Noninvasive Follicular Variant of Papillary Thyroid Carcinoma and the Afirma Gene-Expression Classifier. Thyroid. 2016;26(7):911-5.

4. Jug RC, Datto MB, Jiang XS. Molecular testing for indeterminate thyroid nodules: Performance of the Afirma gene expression classifier and ThyroSeq panel. Cancer Cytopathol. 2018;126(7):471-80.

5. Lithwick-Yanai G, Dromi N, Shtabsky A, Morgenstern S, Strenov Y, Feinmesser M, et al. Multicentre validation of a microRNA-based assay for diagnosing indeterminate thyroid nodules utilising fine needle aspirate smears. J Clin Pathol. 2017;70(6):500-7.

6. Silaghi CA, Lozovanu V, Georgescu CE, Georgescu RD, Susman S, Năsui BA, et al. Thyroseq v3, Afirma GSC, and microRNA Panels Versus Previous Molecular Tests in the Preoperative Diagnosis of Indeterminate Thyroid Nodules: A Systematic Review and Meta-Analysis. Front Endocrinol (Lausanne). 2021;12:649522.
